# Respiratory brain impulse propagation in focal epilepsy

**DOI:** 10.1038/s41598-023-32271-7

**Published:** 2023-03-30

**Authors:** Ahmed Elabasy, Mia Suhonen, Zalan Rajna, Youssef Hosni, Janne Kananen, Johanna Annunen, Hanna Ansakorpi, Vesa Korhonen, Tapio Seppänen, Vesa Kiviniemi

**Affiliations:** 1 Center for Machine Vision and Signal Analysis, University of Oulu, 90014 Oulu, Finland; 2 Diagnostics, Medical Research Center, Oulu University Hospital, 90029 Oulu, Finland; 3 Oulu Functional NeuroImaging, Research Unit of Health Science and Technology, University of Oulu, 90029 Oulu, Finland; 4 Clinical Neurophysiology, Research Unit of Health Science and Technology, University of Oulu, 90029 Oulu, Finland; 5 Research Unit of Clinical Neuroscience, Neurology, University of Oulu, 90029 Oulu, Finland; 6 Neurocenter (Member of ERN EpiCARE), Medical Research Center, Oulu University Hospital, 90029 Oulu, Finland; 7 Biocenter Oulu, University of Oulu, 90014 Oulu, Finland

**Keywords:** Neurology, Epilepsy

## Abstract

Respiratory brain pulsations pertaining to intra-axial hydrodynamic solute transport are markedly altered in focal epilepsy. We used optical flow analysis of ultra-fast functional magnetic resonance imaging (fMRI) data to investigate the velocity characteristics of respiratory brain impulse propagation in patients with focal epilepsy treated with antiseizure medication (ASM) (medicated patients with focal epilepsy; ME, *n* = 23), drug-naïve patients with at least one seizure (DN, *n* = 19) and matched healthy control subjects (HC, *n* = 75). We detected in the two patient groups (ME and DN) several significant alterations in the respiratory brain pulsation propagation velocity, which showed a bidirectional change dominated by a reduction in speed. Furthermore, the respiratory impulses moved more in reversed or incoherent directions in both patient groups vs. the HC group. The speed reductions and directionality changes occurred in specific phases of the respiratory cycle. In conclusion, irrespective of medication status, both patient groups showed incoherent and slower respiratory brain impulses, which may contribute to epileptic brain pathology by hindering brain hydrodynamics.

## Introduction

Epilepsy in its various forms affects roughly 1% of the world population; while the underlying pathology is uncertain, epileptic seizures are often attributed to hypersynchronous bursts of unbalanced inhibitory/excitatory neuronal activity^1^. The majority of epilepsy cases are classified as focal, with an origin in the temporal lobe. The pharmacological targets of most antiseizure medications (ASM) are ion-channels permissive for cation flux across the neuronal membrane; while effective in many cases, fully one third of epilepsy patients fail to respond to pharmacotherapy^2,3^. The phenomenon of pharmacoresistance implies that the underlying pathology need not be a defect in ionic currents per se, but might be also attributable to other factors such as brain hydrodynamics, meaning the cerebrospinal fluid (CSF) circulation and glial-dependent brain waste pathway^4,5^.

A failure in brain water dynamics can create a hyperosmotic interstitial environment that facilitates epileptiform neuronal activity^6^, irrespective of the functional state of neuronal ion channels. Aquaporin (AQP4) water channels, that together with ion channels serve to normalize osmotic balance after transient neuronal activity^6–8^. Indeed, the AQP4 density on hippocampal periarterial astrocytic endfeet is mislocalised by almost 50% in mesial temporal lobe epilepsy patients with hippocampal sclerosis^9,10^, which might predict a decompensation of water dynamics. However, these AQP4 changes were recorded in post-operative resected tissue samples^11^. Non-invasive monitoring of AQP4-driven CSF dynamics in patients living with epilepsy is currently lacking.

The AQP4 channels have recently emerged as pivotal players in maintaining brain solute homeostasis between perivascular CSF and the interstitial fluid (ISF) space in what is termed the glymphatic transport mechanism^4,5^, which enables the export of CSF from the subarachnoid space along periarterial channels, to be further mixed with brain the parenchymal interstitial fluid, and finally exit the brain via the perivenous route^12^. A broad body of evidence shows that physiological brain pulsations are the main drivers of CSF hydrodynamics both at the macroscopic and microscopic levels^13–19^.

Until recently, it has been difficult to non-invasively measure drivers of glymphatic flow in living human brain. However, the fMRI signal power of respiratory brain pulsations increases significantly in cortical regions of slow delta EEG brain activity, which are shown to be characterized by increased interstitial and CSF solute transport^20,21^. Furthermore, there are temporal correlations between respiratory pulse waveforms, CSF oscillations, and electroencephalography (EEG) activity^22,23^. Accordingly, human intracranial electroencephalography electrode studies in patients with intractable epilepsy indicate that the respiratory pulses causally drive electrical local field potentials^24–26^, that may promote depolarization. In line with all these results, the fMRI signal power for respiratory brain pulsations has proved to be abnormally strong, widespread, and hypersynchronous in the brain of human epilepsy patients as compared to matched healthy control subjects (HC)^27–29^. On the other hand, based on our previous work with somewhat differing patient groups, very low frequency (VLF), vasomotor, and cardiovascular frequency bands showed no significant differences between either ME or DN patients contrasted with a HC group^28^.

Arising only in the past few years, ultrafast 3D magnetic-resonance-encephalography (MREG) scanning now allows precise separation of the three known physiological brain impulses, cardiac, respiratory and very low frequencies (VLF) without aliasing^14,16,20^. MREG has already been used extensively in clinical studies^16,20,27–32^.

Recently BOLD signal alone has been noted to present simultaneously both propagating very low frequency waves in addition to functionally connected standing wave patterns^33^. The MREG data similarly shows how respiratory brain pulsations propagate without interruption between CSF spaces and brain tissue^14,16^, where susceptibility weighted blood oxygen level dependent (BOLD) changes dominate in the brain tissue veins^34,35^ and T2* weighted signal-related flow changes dominate in CSF areas devoid of hemoglobin susceptibility effects^36^. Optical flow analysis of the 3D MREG data can be used to quantify the velocity the propagation of such physiological pulsation effects over the whole intracranial space covering both CSF and brain tissue, as speed (**v**_**s**_) and direction (**v**)^37,38^.

We hypothesized that changes in brain CSF hydrodynamic convection of solutes might arise from altered propagation of respiratory pulsations in epileptic brain during the interictal period. To test this hypothesis, we further developed our optical flow analysis^37,38^ to quantify respiratory-driven brain impulses velocity (V_resp_) in 3D brain data in groups of medicated epileptic patients (ME) and drug-naïve seizure patients (DN) as compared to healthy controls (HC). We detected over the entire respiratory cycle multifaceted changes in the propagation of the respiratory induced brain MREG signal and discuss these results with respect to their implications for CSF hydrodynamic convection.

## Materials and methods

### Subjects

We obtained written informed consent from all participants, according to the requirements of the Declaration of Helsinki. All procedures were performed in accordance with the Declaration of Helsinki guidelines. One hundred and seventeen subjects participated in this study: 75 HC were recruited in 2012–2019 (aged 34.2 ± 13.8 years, 38 women). The 42 patients included 23 ME patients with focal epilepsy recruited between 2012 and 2020 (aged 34.2 ± 9.9 years, 15 women), and 19 DN patients recruited between 2016 and 2020, who had experienced at least one seizure, with suspicion of focal epilepsy (aged 36.5 ± 15.9 years, 4 women). All participants were scanned in the Oulu University Hospital. The patients’ demographic data are presented in Table 1. This study was approved by the Regional Ethics Committee of the Northern Ostrobothnia Hospital District. All subjects were instructed to lie quietly in the scanner with their eyes open fixating at a cross on the screen.

**Table 1:** Characteristics of the patients.

Subject	Age (years)	Gender	Duration from diagnosis (years)	Diagnosis	MRI finding	Group
1	32	M	8	TLE bilateral	Hippocampal oedema R	ME
2	38	F	10	TLE	HS R	ME
3	26	F	19	FLE	Normal	ME
4	52	F	4	TLE L	FCD L T	ME
5	36	M	6	TLE L	Arachnoid cyst	ME
6	53	M	13	TLE R	DVA/Telangiectasia R	ME
7	22	F	12	TLE R	FCD R T	ME
8	25	F	11	TLE L	FCD L	ME
9	35	F	12	TLE R	Normal	ME
10	26	F	7	TLE R	Arachnoid cyst R Pituitary microadenoma RFCD R	ME
11	39	F	16	TLE	Normal	ME
12	35	F	17	TLE	Normal	ME
13	26	F	4	TLE R	Normal	ME
14	43	F	4	TLE R	Normal	ME
15	26	M	11	TLE R	Normal	ME
16	36	F	1 month	TLE	Normal	ME
17	43	F	33	TLE	Small aneurysm L	ME
18	17	F	4	TLE	Normal	ME
19	26	M	6	TLE L	Heterotopia L T	ME
20	20	M	2	TEL R	Normal	ME
21	49	F	38	Focal epilepsy	Normal	ME
22	41	M	3 months	TLE L	Normal	ME
23	40	M	4 months	TLE	Normal	ME
24	16	M	4	TLE L	Normal	DN
25	41	M	10	TLE L	Normal	DN
26	39	M	1	TLE	Normal	DN
27	17	M	One seizure	No diagnosis	Normal	DN
28	30	M	3 months	Focal epilepsy	Normal	DN
29	60	F	One seizure	No diagnosis	DNET susp	DN
30	49	M	2	TLE	Temporal vascular degeneration L	DN
31	28	M	One seizure	No diagnosis	Normal	DN
32	21	F	2 months	Focal epilepsy	Normal	DN
33	62	M	2 months	Focal epilepsy	Normal	DN
34	64	M	2 months	TLE	FCD R	DN
35	23	F	No info	Focal epilepsy	FCD L	DN
36	22	M	6	No diagnosis	Normal	DN
37	57	M	One seizure	No diagnosis	Normal	DN
38	25	M	One seizure	No diagnosis	Normal	DN
39	25	M	One seizure	No diagnosis	Normal	DN
40	53	F	One seizure	No diagnosis	Normal	DN
41	37	M	One seizure	No diagnosis	MTS L suspected	DN
42	24	M	One seizure	No diagnosis	Normal	DN

*DVA* developmental venous anomaly, *F* female, *FCD* focal cortical dysplasia, *FLE* frontal lobe epilepsy, *HS* hippocampal sclerosis, *L* left, *M* male, *MTS* mesiotemporal sclerosis, *R* right, *T* temporal, *TLE* temporal lobe epilepsy, *DNET* dysembryoplastic neuroepithelial tumour.

Of the 19 DN seizure patients, seven were primarily diagnosed with epilepsy at the time of scanning and two were later diagnosed with epilepsy in the follow up, such that altogether nine (47%) were diagnosed. According to anamnesis, some among the remaining DN group had experienced epileptic symptoms long before their diagnosis. The remainder of the DN group were without certain diagnosis, and the etiology of their seizure remains unknown. Two patients had been scanned before and after onset of medication: DN subjects 25 and 26, and ME subjects 22 and 23.

### Data acquisition

All subjects were scanned using a Siemens 3 T SKYRA with a 32-channel head coil for an MREG ultrafast fMRI sequence^39^, repetition time = 100 ms, echo time = 36 ms, flip angle = 25°, 3D reconstruction matrix = 64 × 64 × 64 and slab thickness = 192 mm. The data were reconstructed by the L2‐Tikhonov regularization (lambda = 0.1) using the L‐curve method.

### Data preprocessing

MREG data were preprocessed using the FSL pipeline^40^. In order to reach the BOLD signal steady state, we removed 200-time points from the beginning. In total, 2800 3D volumes were analyzed for every subject. Data were highpass-filtered with a cutoff frequency of 0.008 Hz. Head motion was corrected using FSL 5.08 MCFLIRT software. Brain extraction was carried out using FSL 5.08 BET software. Spatial smoothing was carried out with fslmaths 5‐mm FWHM Gaussian kernel. MREG data were registered into 3 mm MNI space using the standard T1 152 MNI brain FSL 5.08 FLIRT software. The MREG data is then band-pass filtered for extracting respiratory band (0.15–0.5 Hz) using AFNI 19.30.01 3dTproject software.

### Analyzing respiratory brain impulses

The MREG signal sampled at 10 Hz in this study reflects combined intracranial effects of CSF and venous flow dynamics across the respiratory cycle, where (according to the Monro–Kellie doctrine) the sum of intracranial brain fluids, i.e. the CSF/ISF and blood volume, is constant over time. Respiration drives the flow and determines the quantities of venous blood and the rate of CSF movement^15,41^. Previous MRI phase imaging studies indicated that inhalation increases caudo-cranial CSF inflow from the spinal canal into the prepontine cistern and ventricles, whereas exhalation reverses the direction cranio-caudal direction driven by the spinal epidural blood flow and pressure effects from respiratory motion^15,41–43^. Simultaneously, inspiration also draws venous blood from the brain into the thorax driven by the negative intrathoracic pressure, which reduces the volume of blood in the cerebral veins^15,41–43^.

The Monro-Kellie doctrine requires that CSF should reciprocally fill the perivenous and other intracranial CSF spaces, since deoxygenated venous blood flows out during inhalation^42,44^. The volume drop of deoxygenated paramagnetic blood inside a given brain voxel decreases water proton dephasing, which is detectable as an increase in T2* weighted blood-oxygen-level-dependent (BOLD) signal upon inspiration. Conversely, exhalation increases the intrathoracic pressure, which causes the epidural spinal and intracranial veins to balloon with deoxygenated blood. As the venous blood accumulates, the CSF drains from perivascular and other intracranial CSF spaces into the subarachnoid space and finally exits the cranium. The decreasing CSF and increasing venous blood volumes independently reduce the BOLD signal, albeit the effect of venous deoxyHb concentration outweighs clearly the perivenous CSF water proton concentration change^14–16,45,46^.

### QPP analysis

A quasi-periodic pattern (QPP) is any complex pattern that has some degree of repetition, but is difficult to describe mathematically due to the lack of simple equations for its description. We used a QPP detection algorithm that employs sliding window correlation and peak detection^16,47^. The algorithm works first divides the MREG time series into non-overlapping segments of fixed length (40 time points/4 s, average length of a respiratory signal). This is necessary because QPPs are often transient and arising at irregular intervals. Next, the algorithm constructs an initial template by applying a window length corresponding to the first 40 time points. For every such segment, a sliding window is then used to compute the cross-correlation between it and the template. The resulting correlation coefficients establish a correlation matrix, which is searched for peaks that are indicative of QPPs. Here, a peak is defined as a local maximum in the correlation matrix that exceeds an adaptive threshold. Once a peak is identified, the algorithm identifies the set of segments that contributed to the peak, which together form a candidate QPP. The candidate QPP is then checked for consistency of the period across segments, duration, and correlation with other QPPs. If it satisfies these criteria, it is accepted as a genuine QPP. We produce subject-specific, normalized Z-scored QPP maps, using MATLAB circshift to ensure that individual QPP maps were all in the same phase. Finally, we produced a population mean QPP map for the HC group, consisting of 60 time points (6 s).

### Optical flow analysis

Sleep increases the power of respiratory pulsations as well as vasomotor and cardiovascular pulsations^20^. Forced voluntary breathing makes respiratory brain pulsations a dominant driving force for CSF flow over cardiac pulsations in awake subjects^42^. Patients with epilepsy have shown increased power of only in the respiratory pulsations compared to healthy controls despite similar non-forced *ad lib* breathing^28^.

**Figure 1:**
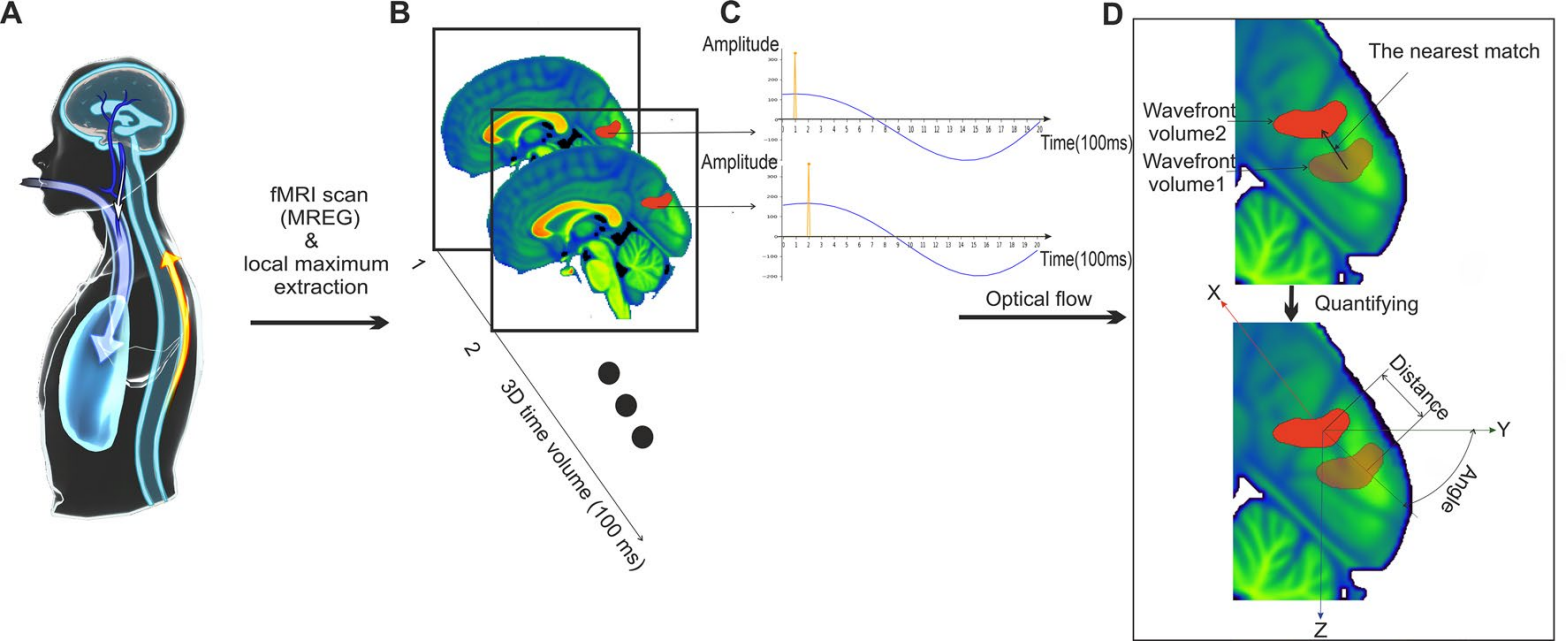
Optical flow analysis demonstration during inhalation. (**A**) An inspiration increases the MREG BOLD signal to a peak as venous blood is drawn into the thorax and CSF flows into the brain. (**B**) the inspiratory signal peak propagates through the brain tissue in waves, which can be detected by the signal peak detector. (**C**) as the respiratory wavefront propagates, the corresponding signal peak in neighboring regions appears with a measurable time dely. (**D**), optical flow analysis can track magnitude **v**_**s**_ and direction **v** of the propagation velocity of the respiratory brain impulse V_resp_ wavefront between consecutive 3D time volumes.

The tracking of the movement of respiration-induced brain signal changes may in principle reveal CSF hydrodynamics at the macroscopic level, which is obtainable non-invasively and without Gadolinium contrast. The 3D optical flow analysis using the Lucas-Kanade multiresolution pyramid offers an efficient way to track the cardiac impulses within the brain^37^. The resolution pyramid approach can accommodate large displacements and improves the accuracy of optical flow speed estimation. In applying the Lucas-Kanade algorithm with resolution pyramid, we first extract the local extrema and minima of the respiratory MREG signal pulse to be the features we track over the brain. Next, we down-sample every frame with these features to create a sequence of lower resolution images. Each subsequent level of the pyramid is created by applying a Gaussian filter to the previous level and then down-sampling it. The number of levels in the pyramid and the degree of down-sampling depend on the size of the image; here we used the third level. We assigned a set of initial motion vectors to each pixel in the highest resolution image. These initial motion vectors could be set to zero or estimated from the previous frame. Next, starting from the lowest resolution image, we apply the Lucas-Kanade algorithm to estimate the motion vectors for each pixel by solving a set of linear equations that relate the pixel intensity values in two consecutive frames. The algorithm uses a window or patch of two voxels surrounding each pixel to compute the local gradient of the image intensity. The motion vector is then estimated as the weighted sum of the gradients within the patch, where the weights are determined by solving the linear equations using the least-squares method. Finally, we up-sample the estimated motion vectors to initialize the motion vectors at the next higher resolution level. This process continues iteratively until the motion vectors have been estimated for all levels in the pyramid. As seen in Fig. 1, applying this procedure on the respiration signal frequency band (0.15–0.5 Hz), quantifies separately the parameters **v** and **v**_**s**_, and their composite, total V_resp_^38^. In particularly V_resp_ is calculated as,

1 $${V}_{resp}={{\varvec{v}}}_{{\varvec{s}}}\underset{\_}{{\varvec{v}}}={{\varvec{v}}}_{{\varvec{s}}{\varvec{x}}}\underset{\_}{{\varvec{i}}}\boldsymbol{ }+\boldsymbol{ }{{\varvec{v}}}_{{\varvec{s}}{\varvec{y}}}\underset{\_}{{\varvec{j}}}\boldsymbol{ }+\boldsymbol{ }{{\varvec{v}}}_{{\varvec{s}}{\varvec{z}}}\underset{\_}{{\varvec{k}}}$$

where **v**ϵℝ^3^, and **i, j, k** are the three standard unit vectors in the X, Y, and Z directions, respectively. In this study, we concentrate on the velocity and its change across the cycle.

### Cycle randomise test

This variant of the Monte-Carlo method differs from the TFCE_FSL randomise test in that it permutes a statistic of respiratory cycles instead of labels of voxels, and in that it uses the harmonic mean correction method. First, we segmented the velocity data to their corresponding respiratory cycles using a central voxel within the fourth ventricle, a position that is significantly affected by respiration^22^. We applied 50,000 permutations, each extracted by selecting a random cycle from a given patient, without any repetition of the same sequence, and then calculated the mean for those cycles passing through a Welch two-sample t-test. Therefore, each test has completely independent data, which results in no biasing from data dependency^48–50^. Although the tests are data dependency-free, the results are dependent in the sense that they represent the same subjects in every t-test result, which calls for a method for combining and correcting the results of the t-tests. The harmonic mean, which is a commonly applied approach for combining dependent data, can be corrected using the method presented in Table 2 of^51^, such that the usual threshold of *p* < 0.05 becomes *p* < 0.03. We applied the cycle randomize test on the parameter of flow velocity directly. However, to find the reversed directions, we applied this method separately on the voxels’ positive and negative signs in each axis (X, Y and Z), and then computed the maximum *p* value in 3D. Any finding of reversed directions represents a reversed propagation of the respiratory signal in the brain of patients with epilepsy relative to the HC group in at least one direction.

### Cycle velocity dynamic analysis

As noted above, we segmented the velocity data to inhalation and exhalation phases using a central voxel in the fourth ventricle^22^. Then, we resampled the velocity data for speed and the displacement in every direction separately for each phase to produce thirty sequential frames/phase (across a single inhalation/exhalation cycle for every subject) using the Fourier method provided in SciPy (v1.7.1, *scipy.signal.resample*). Here, all respiratory phases across subjects have the same duration, which allows frame-by-frame tracking of changes in group-level analysis. The result of this step gives resampled V_resp_, **v**_**s**_, **v**_**sx**_, **v**_**sy**_, and **v**_**sz**_, each of which components has 60 frames/subject. Next, we computed the frame-wise mean of each respiration phase to produce cyclic velocity maps for every subject. Finally, we calculated the group mean maps in a frame-wise manner, and then used the FSL-TFCE randomize test (5000 permutations) to compute statistical differences between every resampled frame in HC versus the two patient groups.

Applying this method allows monitoring separately the parameters of velocity magnitude and direction differences for each frame over the resampled respiration velocity cycle. We applied this method to the positive and negative signs separately along the three axes (X, Y and Z) and we then computed the maximum *p* value resulting for the three dimensions. Here, any result of reversed directions represents reversed propagation of the respiratory brain signal in a patient group relative to the HCs in at least in one direction. We included the framewise displacements as covariates to reduce effect of head motion on this procedure.

### Mutual information analysis

After extracting resampled V_resp_, we compared the patient groups to HC using mutual information, which is a measure of similarity between images and signals^52^, using the software Scikit learn 0.21.3 (sklearn.feature_selection.mutual_info_regression). First, we ordered the data to compare each patient’s respiratory mean cycle flow information to the respiratory mean cycle flow information for the entire HC group. Then we computed the mean mutual information values of the ME and DN patient groups separately. The result of this analysis shows how dissimilar/independent the patients’ V_resp_ information are from the V_resp_ HC group in whole white matter (WM), grey matter (GM), and CSF. The scale of the mutual information is ϵ [0,∞[, where a 0 scale means totally dissimilar/independent and ∞ means perfect similarity/dependency. The infinity value is a main disadvantage of mutual information analysis. However, we are not concerned with absolute values, but rather the relative similarity of each patient group compared to the HC group, such that the greater the deviation of a patient group from the zero value, the less it is similar to the HC group.

### The STD of direction of velocity

After extracting resampled V_resp_ data, we computed the standard deviation (STD) for **v** from spherical coordinates of every subject in the study. Then, we computed the mean value of STD from all brain voxels of every subject, and compared the means of the ME and DN groups with the HC group using unpaired two-sample t-test in the whole volumes for GM, WM, and CSF.

## Results

### Optical flow analysis of respiratory MREG signal

**Figure 2:**
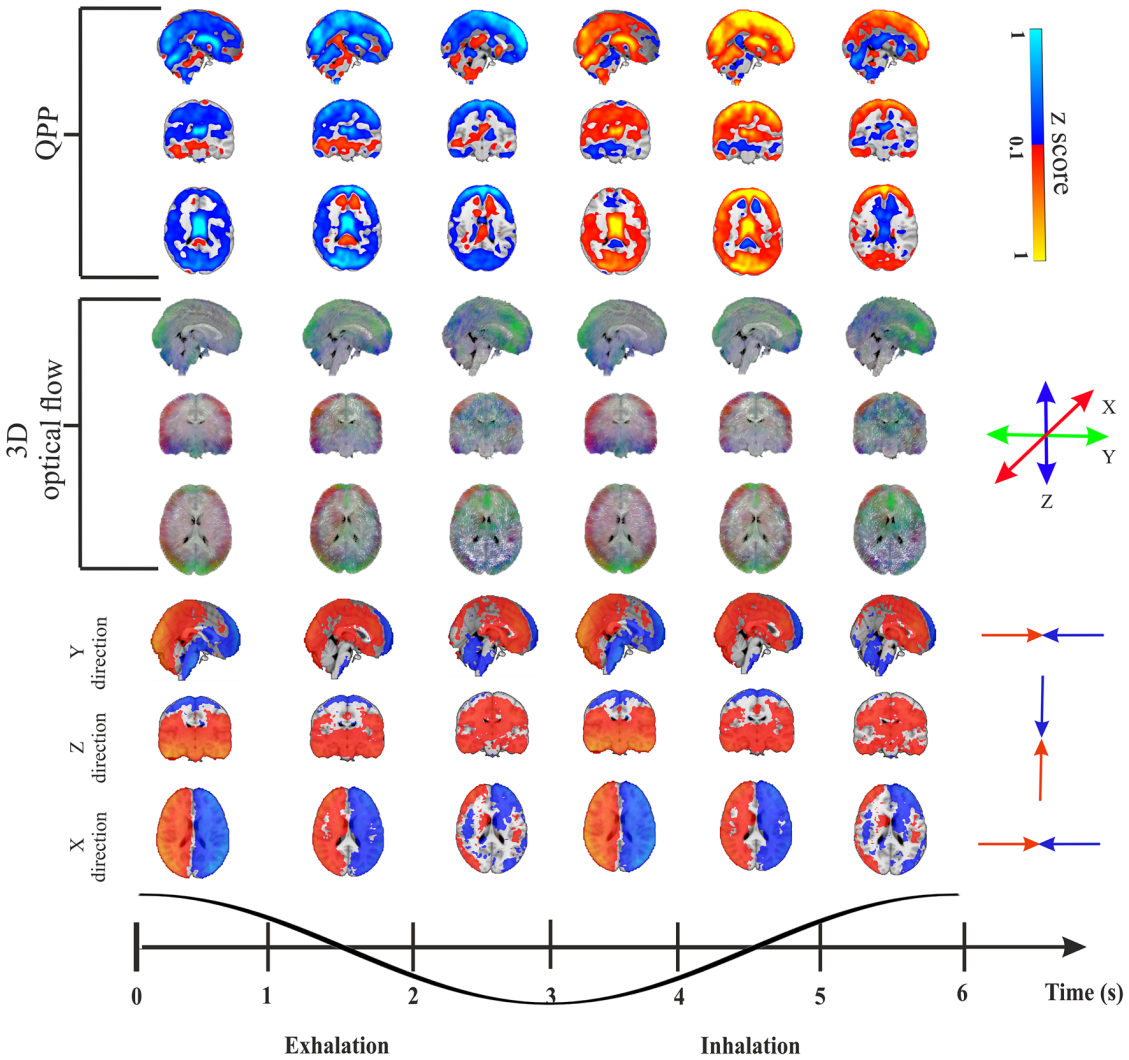
Optical flow analysis to produce mean velocity maps over entire respiratory cycle. (Upper section), optical flow analysis of the input mean data (HC, *n* = 75) of MREG BOLD signal intensity reflecting the QPP respiratory impulse propagation in healthy brain across the respiratory cycle, which is segmented into six epochs from exhalation to inhalation. (Middle section), mean 3D optical flow results from following the respiratory impulse peaks yield combined speed, magnitude, and directionality vectors in standard MNI space. The 3D optical flow result indicates in 3D the [X, Y, Z] directions in [Red, Green, Blue, respectively] colors for the impulse as it propagates through the brain. (Lower section), a 3D directional rendering of impulse vectors shown separately in X, Y and Z directions in opposing (red/blue) directions across the respiratory cycle. *All the results are also illustrated dynamically in Supplementary Videos. The result of the optical flow mean analysis and the velocity components is presented in Supplementary Video 1. The result of the optical flow mean analysis for HC, ME and DN is presented in Supplementary Video 2.

As shown earlier, respiration induces a propagating signal wavefront passing through the brain with cyclically during each respiration, as revealed in bandpassed MREG signal data^14,16^. Figure 2 illustrates mean respiratory signal changes at six phases over the cycle, where the lower part of the figure illustrates mean periodic velocity maps from optical flow maps of a group of 75 healthy controls.

The moving respiratory wavefront in brain has velocity V_resp_ = **v**_**s**_**v**, as in Eq. (1), where **v**_**s**_ is the magnitude of the wave propagation displacement/time and **v** is the unit vector of the displacement direction. As depicted in Fig. 2, the QPP of the mean input respiratory MREG signal shows that the magnitude of BOLD signal changed in opposing ways across the respiratory cycle. We observed that the magnitude of V_resp_ was mostly symmetric between inhalation and exhalation phases, thus indicating *similar* flow directions across both phases (see Fig. 2). Notably, the flow in cortical GM is more along sinuses, while in deeper WM the waves are directed along WM veins towards the brain center, c.f. Fig. 2 3D optical flow. At the end of inhalation and exhalation phases, there seem to be short periods of opposing reflux flow in the middle of the WM. In CSF spaces, however, the respiratory pulsations circulate in directions that oppose the cortical venous flow directions. Anatomically, the respiratory impulses were symmetric and centripetal in the axial (XY) plane directed towards the brain center. But in the interhemispheric fissure in the sagittal plane the rostrocaudal Z-direction flow introduced a marked periodic asymmetry in the impulse, which spread inwards as a rotatory flow pattern in relation to transient net flow changes.

### Mean velocity changes in focal epilepsy

**Figure 3:**
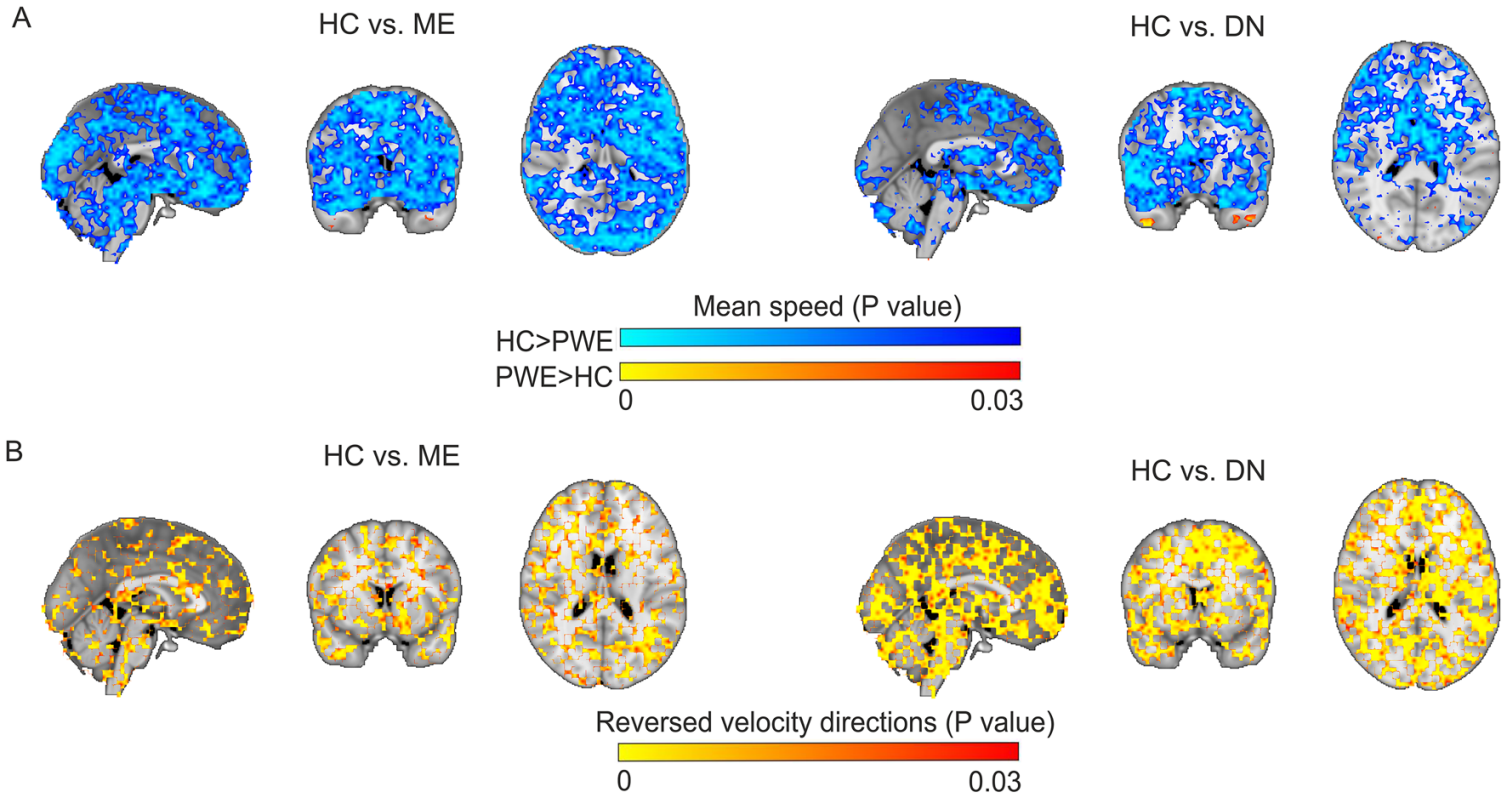
Alterations in mean velocity of respiratory brain impulse. (**A**) statistical difference maps of the respiratory impulse propagation speed v_s_ between the ME group (*n* = 23) vs. HC group (*n* = 75) (left), and the DN group (*n* = 19) vs. HC group (*n* = *75*) (right). The statistical differences (cycle randomise test, harmonic mean correction, *p* < 0.03) show a significant reduction in **v**_**s**_ over most parts of the cerebrum and cerebellum as well as brainstem in both patient groups. (**B**) statistical comparison maps of reversed **v** of the respiratory impulse propagation between the ME and HC groups (left), and between the DN and HC groups (right). The mean **v** is widely reversed over the whole respiratory cycle in the ME and DN groups in patchy areas extending throughout the brain (cycle randomise test, harmonic mean correction, *p* < 0.03).

In order to find out whether the respiratory-induced CSF/ISF flow is altered in patients with focal epilepsy, we compared statistically the mean of individual MREG optical flow results of the respiratory pulse propagation velocity information between the complete groups of 23 ME and 19 DN patients and the 75 HC. The magnitude of respiratory pulse propagation speed **v**_**s**_ differed significantly in the ME and DN groups (Fig. 3). The **v**_**s**_ had a very widespread reduction (mean vs mean, cycle randomise test, harmonic mean correction, *p* < 0.03), predominantly on the left side, and encompassing all lobes of the cerebral cortex for the ME group, although there was a small area of increased speed in the left interior temporal lobe of the ME group. Overall, there was a reduction in CSF/ISF flow speed in the ME group (Fig. 3A).

As the respiratory pulse speed was reduced in the patients, this raised the question of whether the direction of the CSF/ISF flow could also be altered. Therefore, we analyzed the mean map of **v** of each group, which revealed an overall diffusively reversal of the mean of **v** encompassing the whole brain in the ME and DN groups compared to the HC group (Fig. 3B). Taken together, these results imply that the CSF/ISF respiratory flow is stagnated in epileptic brain data, as evidenced by the dominantly reversed and reduced propagation velocity.

These changes in speed and directionality could be due to anti-seizure medication (ASM). However, our corresponding analysis in the DN with seizures showed similar decreases in **v**_**s**_ and reversal of **v** as seen in the treated group of patients with epilepsy diagnosis. The overlaps of the DN group mean signal with ME findings were most pronounced in frontal and temporal lobes (Fig. 3). Also, there was a small increase in the **v**_**s**_ in the bilateral temporal lobes. The overall directionality investigation shows a more widespread reversal of **v** over most parts of the brain, as shown in Fig. 3B, which suggests that medication may have resulted a partial rescue of the hydrodynamic parameters in patients.

### Do changes in respiratory impulse propagation depend on the respiratory phase?

**Figure 4:**
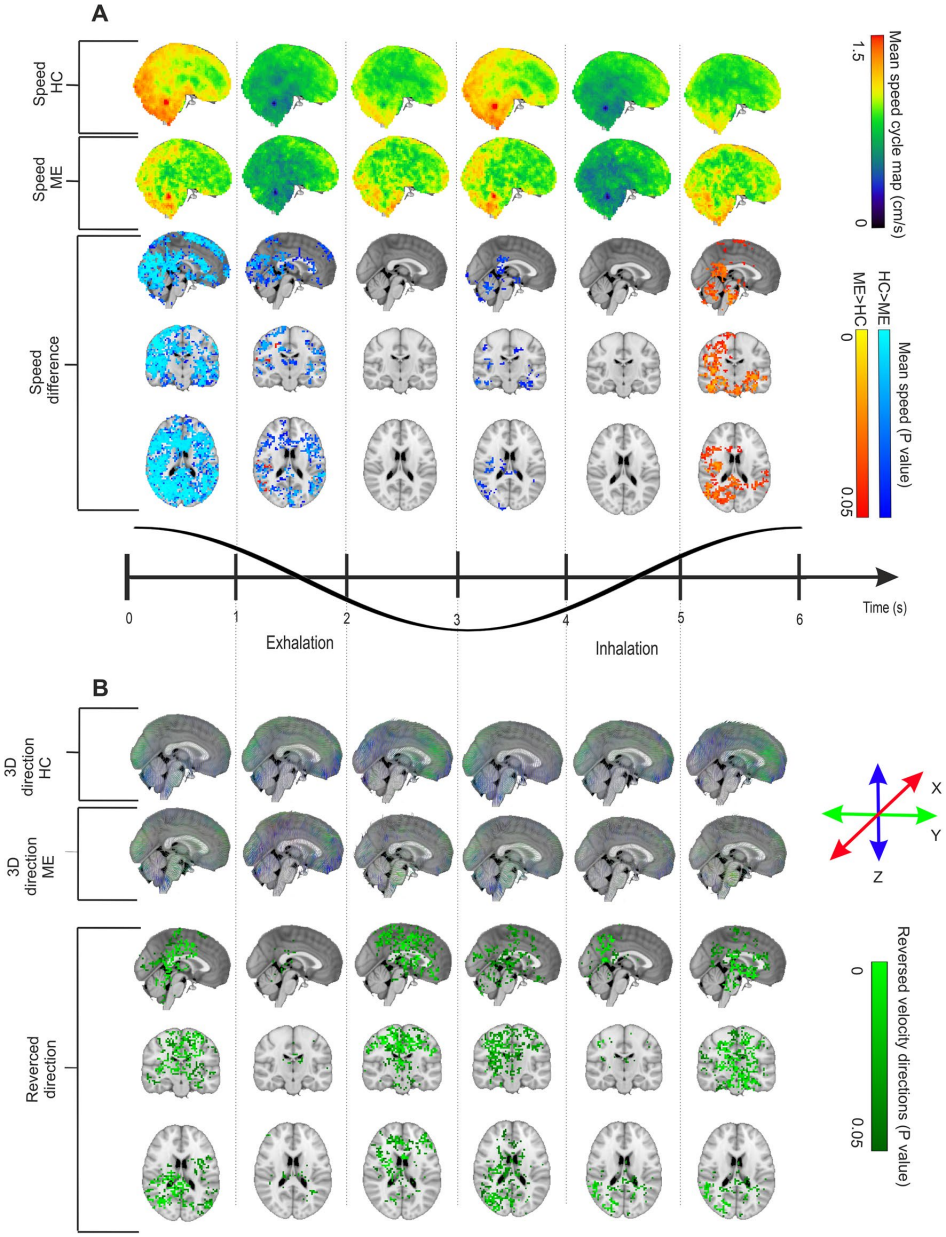
Comparison between HC and ME groups in mean V_resp_ brain impulse over an entire respiratory cycle. (**A**) mean magnitude maps of respiratory impulse **v**_**s**_ shown across six phases of the inhalation/exhalation cycle for the HC group (*n* = 75) (top) and the ME group (*n* = 23) (middle). At the bottom, findings in the X, Y, and Z directions illustrate significant (FSL *randomise*, family-wise-error-rate (FWER) correction, *p* < 0.05) group spatial differences between the respective respiratory cycle phase means, with the most significantly reduced **v**_**s**_ in ME patients during the early part of exhalation. We see a small phase of increased **v**_**s**_ at the late inspiratory phase just preceding before the large exhalation-associated **v**_**s**_ decrease. (**B**) mean respiratory impulse velocity 3D direction maps showing **v** in across the six phases of inhalation/exhalation cycle averaged over the respiratory cycle, of HC group (top) and ME group (middle). At the bottom, we see (FSL *randomise*, FWER correction, *p* < 0.05) differences between the respective respiratory cycle phase averages. Each phase shows a marked reversal of V_resp_, which vanishes during the middle phases both of inhalation and exhalation. Supplementary Videos 3 (**v**_**s**_) and 4 (**v**) visualize the dynamic nature of the V_resp_ pathology in epilepsy.

As the V_resp_ significantly differed in the seizure groups compared to HC, we set out to investigate if these changes in the respiratory propagation were dependent upon a certain phase of the respiratory cycle. We therefore performed a dynamic phased analysis by tracking the V_resp_ of the respiratory BOLD signal and group-level differences across the six epochs of the respiratory cycle, namely three exhalation phases and three inhalation phases, as seen in Fig. 4. The mean **v**_**s**_ was 0.954 cm/s (range 0–2.331 cm/s) for HC, versus 0.918 cm/s (range 0–2.085 cm/s) for the ME group. In Fig. 4A, for illustration purposes the scaling is set to the range of 0–1.5 cm/s.

We note that there were brain regions showing significant (*p* < 0.05) relative increases (ME group > HC group) and decreases (ME group < HC group) in **v**_**s**_ over the respiratory cycle. The most widespread and significant decrease of **v**_**s**_ was detected during early exhalation, with a brain-wide extension sparing only the central pontine and inferior cerebellar structures, c.f. Figure 4A. On the other hand, at the peak of inhalation, **v**_**s**_ as increased in the right posterior hemisphere and in temporomesial and infratentorial structures of the ME group. Unexpectedly, we observed right sided dominance in V_resp_ alterations in both patient groups. This seems to be a real group effect rather than arising from technical issue such as scanner artifact, as all subjects were scanned using the same scanner.

The **v** changes seen across the respiratory cycle indicate that the reversal of V_resp_ occurs in coincidence with the peaks both of inspiration and exhalation, Fig. 4B. The **v** changes tended to localize over and near the ventricular system, but also involved WM and GM, as well as the CSF-filled cerebral ventricles. The reversed **v** changes seem to proceed in a rostrocaudal direction in repeated cycles during the reversal from exhaling to inhaling, c.f. axial slices at the bottom in Fig. 4B.

### Drug-naïve patients with at least one seizure present similar results

We have hither presented findings in the ME group, which may have been confounded by effects of ASM. Therefore, we conducted parallel studies in a group of patients with a recent seizure, but 53% of them without epilepsy diagnosis and all of them without ASM treatment (DN group). For overall visual comparison of the V_resp_ between the HC, ME, and DN groups, we refer to Supplementary Video 2. The mean **v**_**s**_ for the DN over the whole respiratory cycle was found to be 0.93 cm/s (range 0–2.517 cm/s), thus falling within the range for ME and HC groups.

**Figure 5:**
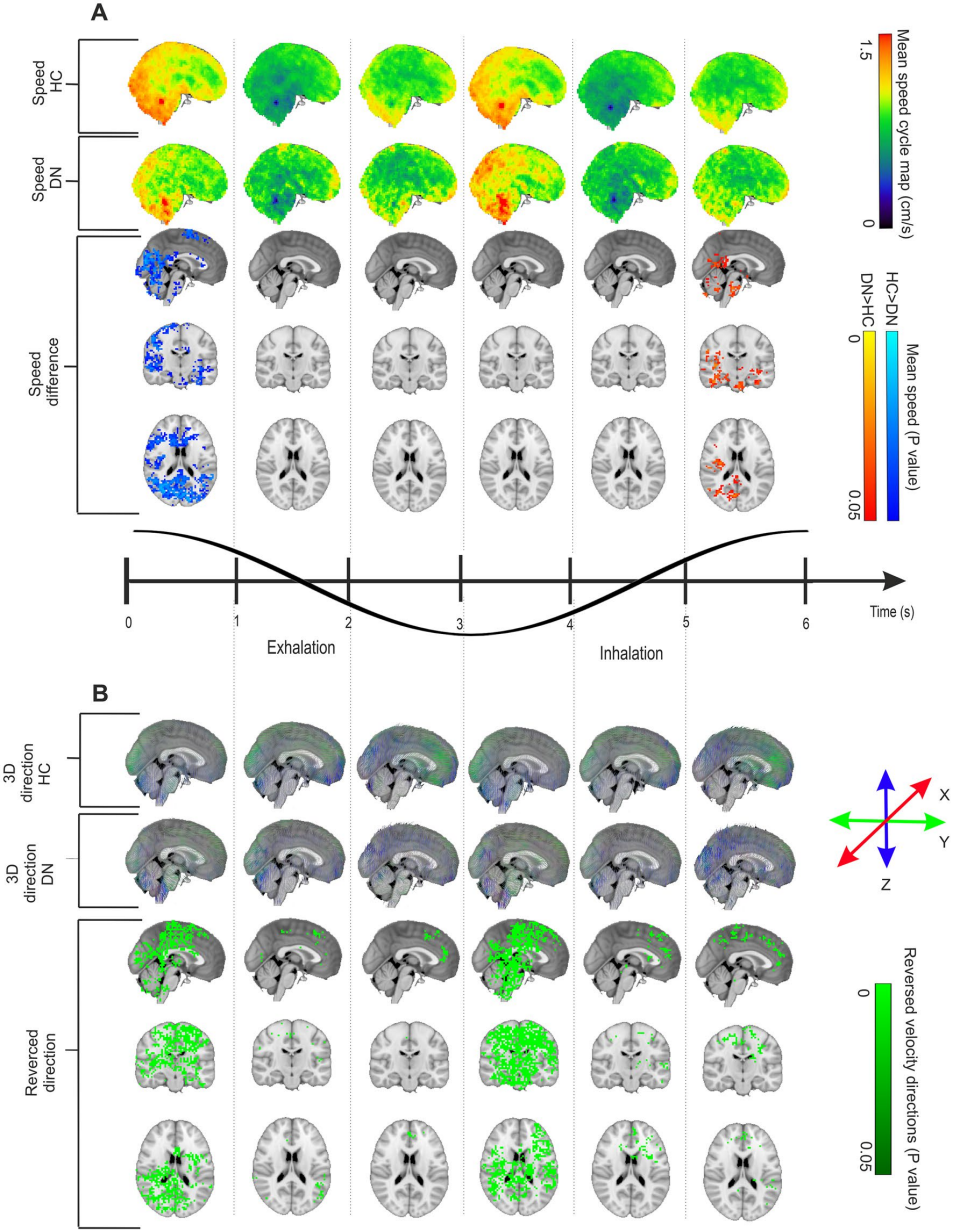
Comparison between HC and DN groups regarding mean voxelwise V_resp_ brain impulse over an entire respiratory cycle. (**A**) mean **v**_**s**_ maps of respiratory impulse shown across six phases across the inhalation/exhalation cycle, HC group (*n* = 75) (top) and DN group (*n* = 19) (middle). At the bottom, we see 3-direction (X, Y, and Z) maps illustrating significant (FSL *randomise*, FWER correction, *p* < 0.05) group spatial differences between respective respiratory cycle phase averages. Similar to the ME data in Fig. 4, the most significant group difference was the relative reduction of **v**_**s**_ in the DN group during early exhalation, preceded by a spatially small phase of increased **v**_**s**_ in the late inspiratory phase prior to exhaling. (**B**) mean respiratory impulse velocity 3D direction maps shown across the six phases of the inhalation/exhalation cycle averaged over the respiratory cycle for the HC group (top) and DN group (middle). At the bottom, there are the reversed **v** brain direction differences (FSL *randomise*, FWER correction, *p* < 0.05) between respective respiratory cycle phase averages. Reversed **v** was detected in large mainly cortical areas during the early phases both of exhalation and inhalation, but lasting over a shorter period than in the ME group. Supplementary Videos 5 (**v**_**s**_) and 6 (**v**) the dynamic nature of the V_resp_ pathology in untreated DN. Directional instability in epileptic brain respiratory impulse propagation.

In line with findings in the ME group, the respiratory phase analysis results for the DN group showed similar but less powerful respiratory phase dynamics regarding the **v**_**s**_ and **v**. The reduced **v**_**s**_ occurred similarly during the early exhalation phase, while the increased **v**_**s**_ was observed at the end of the inspiration phase (Fig. 5A). Spatially, the changes were highly similar in the DN group, albeit less widely spread than in the ME group, especially for the **v**_**s**_ increases. Also, the **v**_**s**_ and **v** changes were absent during the intermittent phases, manifesting only over the respiratory peaks in the DN group. The reversed **v** of the DN group relative to the HC group was less widespread across the brain than the corresponding reversal in the ME group. Furthermore, the reversed **v** occurred mostly at the beginning of each respiratory phase (Fig. 5B). The directionality changes are also more spatially focused in the DN group compared to the ME group, although temporally they likewise occurred after exhalatory and inhalation peaks across the cycle. The directionality change was also more intense and focused mainly in one phase in the DN group, c.f. Figure 5B. However, the brain voxels showing significant directionality changes were less widespread in the DN group as compared result than in the ME group.

**Figure 6:**
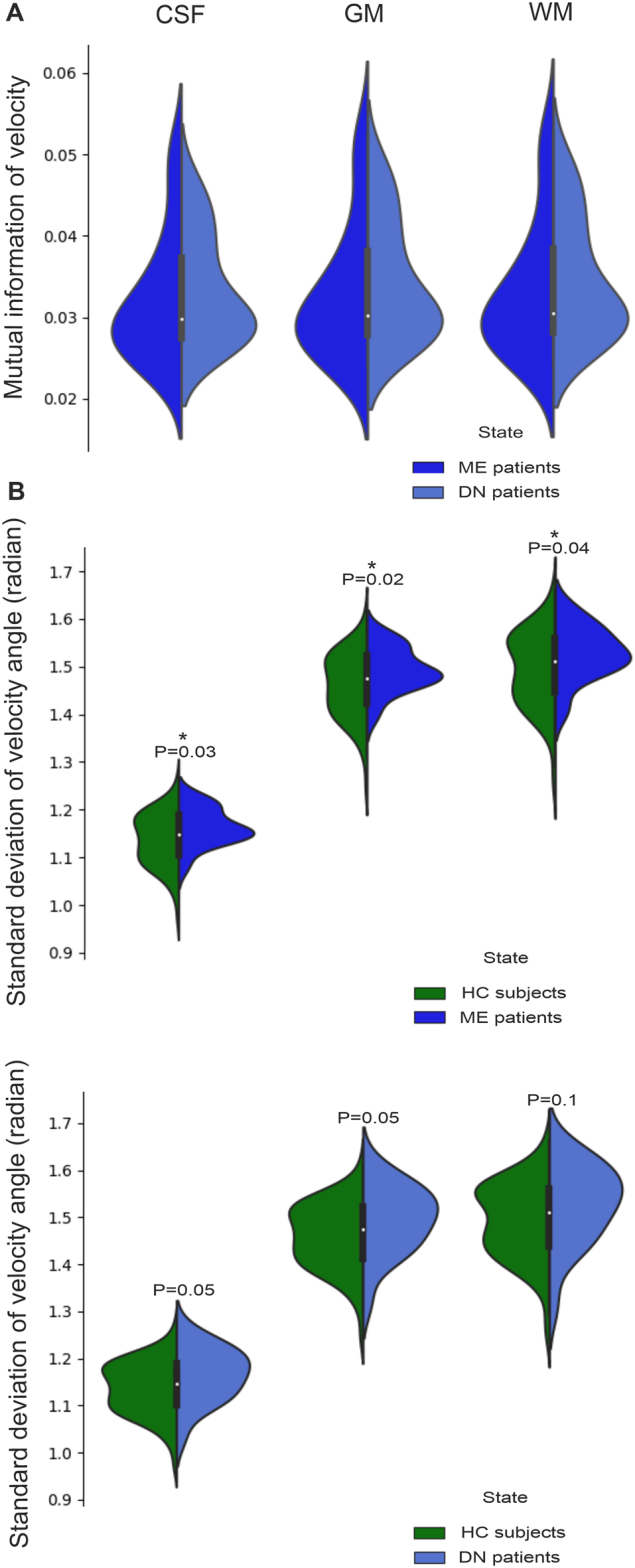
Comparison of the velocity of resampled respiratory MREG signal between ME and DN groups versus the HC group. (**A**) mean mutual information of mean global V_resp_ in (ME, *n* = 23 and DN, *n* = 19) groups relative to the HC group (*n* = 75), shows that the mutual information is very close to zero, i.e. the pulsations are highly divergent in the pooled patients, with the ME group being more divergent than the DN group. (**B**) STD of **v** between ME and DN groups vs. the HC group shows that the ME group had significantly (unpaired two-sample t-test, *p* < 0.05) more deviation from the mean flow directionality measured in the HC group, whereas, the DN group differed at the margin of significance (unpaired two-sample t-test, about *p* = 0.05).

In examining the directionality result videos, one can see that the directionality of V_resp_ is highly stable and targeted in the brain of HC, while the directionality seems visually more variable over the respiratory cycle in the ME and DN groups (Supplementary Video 1, 2). Thus, we quantified the stability of the V_resp_ in the three groups by mutual information (mutual information V_resp_ map, c.f. Supplementary Fig. 1) and STD of the velocity directionality. This approach revealed that the mutual information of the V_resp_ in the ME and DN groups was nearly zero compared to the HC group, indicating marked dissimilarity in the overall dynamics of V_resp_, where the ME group was less similar to HC group in V_resp_ signal behavior than was the DN group, as shown in Fig. 6A. Furthermore, the temporal STD of the ME and DN groups was significantly higher in the CSF, WM and GM in the ME group (*p* < 0.02–0.04) compared to controls, and nearly significantly higher (*p* = 0.05) in CSF and GM in the DN group compared to the HC group, as shown in Fig. 6B. These results indicate that the reduced net **v**_**s**_ and reversed **v** in the epileptic brain induce markedly more unstable and less coherent V_resp_. To ensure that the STD difference was not due to the differing population size of groups, we performed the same analysis in a contrast of 23 randomly selected HC versus the 23 ME, as shown in Supplementary Fig. 3 and Video 7, and the results were nearly unaffected by the truncated HC group.

### Motion artifacts and physiological frequencies

There was a significant group difference in the framewise displacement in our data (*p* < 0.05). We minimized the effect by using FSL 5.08 MCFLIRT software for data preprocessing, and applied the motion regressors as covariates in the FSL *randomise* test. The means respiratory frequencies were 16.02 ± 2.40 HC, 15.66 ± 2.28 ME, and 16.13 ± 2.20 DN breathing cycles per a minute, which did not differ significantly for in the ME and DN groups relative to HC (*p* = 0.26, *p* = 0.42, respectively).

## Discussion

In our endeavor to assess respiratory driven brain CSF dynamics in ME and DN patients, we used 3D ultra-fast MREG signals over the whole brain to measure the magnitude **v**_**s**_ and direction **v** of respiratory brain pulse propagation velocity, relative to the HC group. We detected marked **v**_**s**_ reductions, and, also reversed **v** both in the ME and DN groups compared to the matched HC group. Intriguingly, the phase of the respiratory cycle had a significant effect on the velocity changes; the detected drop of the **v**_**s**_ in patient groups occurred almost exclusively during the initial phase of expiration, preceded by a relatively smaller increase in **v**_**s**_ in the late inspiratory phase. Also, the direction of the flow **v** was regionally reversed in ME compared to controls over most of the respiratory cycle. We further show that the flow changes are unlikely to be influenced by treatments of patients with ASM, as almost similarly intense differences in **v**_**s**_ and **v** were also detected in the DN group in brain overlapping areas. Based on a visual inspection of the dynamic directionality speed vectors in 3D, we finally analyzed the stability of V_resp_, finding that the **v** was significantly more variable and incoherent in both ME and DN groups compared to the HC group.

We discerned right sided dominance in V_resp_ alterations in both patient groups, which is not readily attributable to technical faults. Seizure onset foci were not consistently lateralized in the patients, some of whom were right-sided, some left-sided, and others of unknown site of seizure origin. Therefore, we can propose that the asymmetry may arise from the net asymmetry of seizure onset zones. Furthermore, this novel respiratory pulsation abnormality occurred over a wide range in power analyses, and did not consistently show a regional distribution, especially in whole group analyses.

### Macroscopical change in CSF dynamics

We have described a new method for non-invasively quantifying respiratory wave-induced brain impulse speed in the human brain.

In control cases, respiratory waves in the brain parenchyma propagate mostly along venous anatomy, tracking cortical veins in the cortex, and in the deep WM, following deeper veins towards central brain regions. This is physiological appropriate, as respiration is the dominant force driving venous blood flow^42,45,53^, such that the associated signal source is predominantly a susceptibility-weighted BOLD signal. However, in the CSF, the pulsations seem to rotate in a manner somewhat opposing ways venous flow, which reflects the Monro-Kellie doctrine of intracranial hydrodynamics.

Based on our T2* weighted MREG signal, we detected a net flow towards the brain. It does not follow that we adhere to a unidirectional CSF circulation theory^54^. However, we do show that flow direction pulsates up and down along the spinal cord, but with net flow up towards the brain. These results are in excellent agreement with previous work^13,44^. In the supine position, the net CSF flow in a forced respiratory cycle is towards the brain. Thus, during forced inspiration, the CSF moves up towards the brain, whereas during forced expiration, the CSF moves downwards to the spinal cord, and upwards to the brain in a watershed pattern with a dividing point at about the level of the heart, i.e. approximately T6. Our phase contrast studies focused on the cerebral aqueduct on a single slice area, and our results are T2* weighted magnitude data extending over the whole brain.

The mean **v**_**s**_ goes up to 2.331 cm/s in the fourth ventricle and around the cerebellar fossa, which are common CSF markers used to capture respiratory signals. The speed range is virtually identical to flow speeds in the spinal canal, which were also as high as 2 cm/s based on phase imaging^13,43,55^. Furthermore, the CSF flow speed measure in the pontine cistern gave a nearly identical speed range compared to findings in a phase contrast imaging study^55^.

The phase and depth of respiration has a well-established connection to epilepsy^27–29^, in contrast to other physiological brain pulsations^28^. In routine EEG diagnostics, hyperventilation reduces epileptic seizure threshold and increases neuronal excitability, and is thus routinely used to provoke seizure activity during recording session in patients with epilepsy, even though the underlying mechanisms remain unknown^56^. The increased power of respiratory brain pulsations detected in epileptic brains may similarly affect the CSF solute transport, alter neuronal excitability thus lowering the seizure threshold^28^, especially given the tendency for both slower and reversed impulse propagation (Figs. 4B and 5B).

In subarachnoid periarterial spaces of the mouse brain, cardiac pulsation is a stronger driver of glymphatic solute transport than is respiration^17^. However, macroscopically in the human brain, the respiratory pulsations are dominating^16^. Furthermore, during forced/yogic abdominal breathing, respiration is the main driving force for CSF dynamics^42,45,53^. Abnormal respiratory patterns may also induce abnormalities in CSF convection, potentially promoting a transition to the ictal state. Indeed, patients with the chronic obstructive pulmonary disease have a higher risk of stroke-related seizures^57,58^. Sleep increases brain CSF clearance^4,5,21^, and accordingly, during sleep the power of all three physiological pulsations in the human also increase, especially vasomotor waves and respiration^20^. Unsurprisingly, chronic sleep-related breathing disturbances have an association with epilepsy^59^. In one study, continuous airway pressure treatment of obstructive sleep apnea helped to reduce the seizure frequency to half in 70% of patients with epilepsy (PWE)^60^.

Intracranial needle electrode studies in humans indicate that the respiratory-related brain pulsations drive neuronal activity, insofar as field potentials track the respiratory cycle^25,26^. In line with these studies, our group has previously shown marked increases in respiratory impulse amplitude, variance, and brain wide synchrony in epileptic brain^27–29^. In the present study we detected bi-phasic changes in the respiratory brain impulse propagation in epileptic brain ME and DN compared to HC, which depended on the position in the respiratory cycle; the changes were dominated by **v**_**s**_ reductions during early exhalation, preceded by a spatially smaller increase at the end of the inhalation phase, Figs. 4, and 5.

The directionality of the pulse propagation **v** in both patient groups showed a large spread of mean reversed velocity patterns, suggesting that the net effect of the CSF pulse convection can be somewhat reduced or indeed stagnated, especially in the ME group (Figs. 4, 5, Supplementary Videos 3, 4). After examining more closely the temporal variability of the magnitude of **v**, it emerged that the flow patterns had much more variability in the patient groups compared to the HC group (Figs. 3 and 6 and Supplementary Vidoes 4–6). The propagation routes for brain tissue CSF impulses may be obstructed in epilepsy, forcing the flow to follow tortuous and diverted routes. The directional variability that we quantified in patients may arise in part from a disbalance in the two sources of respiratory brain pulses: the counteracting venous outflow vs. reciprocal CSF inflow (c.f. the “Materials and methods” section).

Venous wall structures and/or the perivenous CSF space may both be involved in the pathology leading to obstructed flow. In either case, a stagnation of CSF convection in (peri)venous tissue in epileptic brain could explain the finding of reduced **v**_**s**_ and reversed **v** changes in the V_resp_. Furthermore, these results clearly indicate that the incoherent V_resp_ is less efficient in ME and DN groups compared to the HC group, suggesting that the reduced **v**_**s**_ in epilepsy may also partially stem from an increased resistance to CSF flow, again consistent with physical obstruction.

Epilepsy is related to a plethora of diseases, many of which can cause stagnant in (peri)venous structures, e.g., acute trauma, edema and hemorrhage, brain tumors, and post-infectious or traumatic gliotic changes that develop over years^7,61^. All of these pathological processes have in common the propensity to obliterate locally the low-pressure veins, and likewise the surrounding (peri)venous structures. These microscopic changes are not detectable in MRI scans, while in pathological specimens examined ex-vivo have lost patency of the perivascular space due to loss of CSF pressure. Even minimal, microscopic structural damage could hinder the perivenous pulsation that drives CSF/ISF convection, and thus prevent the venous volume pumping that normally occurs during exhalation; furthermore, epileptic activity can promote uptake of blood-born substances like albumin and lactate into CSF secondary to BBB disruption, which might even further hinder CSF flow^62^.

### Microscopical background hypothesis

As stated above, the actual pathology in epilepsy is not always identifiable macroscopically, even with the latest MRI or other diagnostic tools, and the picture is complicated by the multiple predisposing conditions in epilepsy^63^. We suppose that in many cases the pathology may consist of microscopic changes in structures facilitating extracellular ion fluxes that set the extracellular ion concentrations.

Many recent studies support the link between failed brain solute transport and neurodegenerative disease such as Alzheimer’s disease, stroke, and epilepsy^64,65^. Epileptic seizures are a common occurrence in patients with mild Alzheimer’s disease, which is also increasingly being linked to dysfunction of glymphatic solute transport, especially affecting the cardiovascular drivers of the CSF flow^31,38,66,67^.

Experimental findings implicate dysregulation of water and ion homeostasis in the pathology of mesial temporal lobe epilepsy, the most common form of seizure disorders. In the event of mesial temporal lobe sclerosis, there is a complete loss of AQP4 polarization in perivascular astrocyte endfeet, which impairs the interstitial and glial water inflow, leading to the characteristic tissue shrinkage^7,9^, which may itself be a factor promoting seizure generation. Increased extracellular glutamate is also commonly reported in epileptic tissues^7,68^. Alterations of metabolic waste clearance were observed in the brainstem and thalamus in patients with impaired consciousness^69^. Recently, sleep/wake dysregulation was found to be connected to widespread brain pulsation abnormalities in narcolepsy^32^.

### Limitation and further investigations

As we have discussed above, there were significant group differences in the frame-wise displacement between controls and patients, which may have biased the results. In addition to standard Mcflirt motion correction, we used motion regressors as covariates in the FSL-TFCE randomise test to minimize the effects of head motion on our endpoints. In addition, rigid body craniocaudal z-direction head displacement is known to vary over the respiratory cycle^70^. Recently, a second type of elastic brain tissue motion has been described in which 3D brain deformation is linked to cardiorespiratory pulsations^71^. Current motion correction methods are unfit to counter act or correct these types of motion, which presents problem that applies to all brain imaging data, irrespective of the scanning method. Our present result revealed widespread V_resp_ alterations in the brain of the two epilepsy groups. However, there is considerable brain deformation due to respiration, notably in the ventricles and close to the aqueduct of Sylvius. These deformations might have contributed to our observed differences in V_resp_ in the ventricles and the aqueduct. Consequently, we are cautious in our interpretation of findings in these regions, especially vulnerable to motion artefact. The design of our techniques and our insights regarding the V_resp_ direction (c.f. Supplementary Fig. 2) suggest to us that motion artifacts could only have had a minor overall effect in our group analysis.

As likewise seen in the ME group, the DN group also had considerable clinical heterogeneity, which we note as a limitation. Only nine of the 19 DN seizure patients eventually had a definite, EEG-based diagnosis of epilepsy; the remainder had been evaluated by neurologists, who concluded that the occurrence of at least one epileptiform and/or epileptic seizure could not be excluded. Nevertheless, we note that the DN individuals with at least one definite seizure prior to the MREG scan presented multiple significant changes in their respiratory brain pulsations^28,29^, which suggests that the respiration changes could indeed reflect elevated sensitivity to seizure activity, even without meeting the criteria for a clinically manifest epilepsy diagnosis.

Present results lead us to plan further multichannel studies with combined EEG and MREG imaging data to quantify neuronal activity changes related to the respiratory pulse abnormality in the epileptic brain. Also, we plan to analyze changes in functional brain connectivity in epilepsy with respect to the detected respiratory impulse changes. Finally, group-level studies could open new possibilities for a more precise diagnostics at a single subject level.

Summing up the previous studies in this domain, the detected significant V_resp_ alterations in both patient groups may be related to altered interstitial brain tissue CSF/ISF flow. Given the dominating role of respiratory brain pulsations in this process^16^, and considering its important effects on interstitial neuronal activity in both sleep^20^ and sleep/awake dysregulation in narcolepsy^32^, the reduced and more complex respiratory flow changes that we have observed in epilepsy could reflect deficient hydrodynamic solute transport of the brain tissue. Plausibly, this circumstance could sensitize brain tissue to osmotic and electrolytic disturbances promoting epileptic activity. Interstitial water dynamics connected to macroscopic CSF water flow alterations may play a key role in establishing insalubrious local changes in metabolite, electrolyte, and neurotransmitter concentrations, which may together comprise the functional disturbances found in patients with epilepsy, especially in its intractable forms resistant to pharmacological treatments targeting ion channels or the balance of excitatory/inhibitor neurotransmission.

## Supplementary Information

Supplementary Information.

Supplementary Video 1.

Supplementary Video 2.

Supplementary Video 3.

Supplementary Video 4.

Supplementary Video 5.

Supplementary Video 6.

Supplementary Video 7.

## Data Availability

The data are available upon reasonable request: vesa.kiviniemi@oulu.fi.

## Code availability

For 3D optical flow analysis, please contact: Zalan.Rajna@oulu.fi. For the statistical tools, please contact: Ahmed.Elabasy@oulu.fi.
