# Author Correction: **Respiratory brain impulse propagation in focal epilepsy**

**DOI:** 10.1038/s41598-023-34268-8

**Published:** 2023-05-09

**Authors:** Ahmed Elabasy, Mia Suhonen, Zalan Rajna, Youssef Hosni, Janne Kananen, Johanna Annunen, Hanna Ansakorpi, Vesa Korhonen, Tapio Seppänen, Vesa Kiviniemi

**Affiliations:** 1 Center for Machine Vision and Signal Analysis, University of Oulu, 90014 Oulu, Finland; 2 Diagnostics, Medical Research Center, Oulu University Hospital, 90029 Oulu, Finland; 3 Oulu Functional NeuroImaging, Research Unit of Health Science and Technology, University of Oulu, 90029 Oulu, Finland; 4 Clinical Neurophysiology, Research Unit of Health Science and Technology, University of Oulu, 90029 Oulu, Finland; 5 Research Unit of Clinical Neuroscience, Neurology, University of Oulu, 90029 Oulu, Finland; 6 Neurocenter (Member of ERN EpiCARE), Medical Research Center, Oulu University Hospital, 90029 Oulu, Finland; 7 Biocenter Oulu, University of Oulu, 90014 Oulu, Finland

Correction to: *Scientific Reports* https://doi.org/10.1038/s41598-023-32271-7, published online 30 March 2023

The original version of this Article contained errors in the Affiliations.

Affiliation 2 was incorrectly given as ‘Medical Imaging, Physics and Technology, University of Oulu, 90029 Oulu, Finland’. The correct affiliation is listed below.

Diagnostics, Medical Research Center, Oulu University Hospital, 90029 Oulu, Finland.

Affiliation 7 was incorrectly given as ‘MRC, Oulu University Hospital, 90029 Oulu, Finland’. The correct affiliation is listed below.

Biocenter Oulu, University of Oulu, 90014 Oulu, Finland.

Additionally, affiliation 3 was omitted from Zalan Rajna and affiliations 3 and 7 were omitted from Vesa Kiviniemi.

Finally, Janne Kananen was incorrectly affiliated with ‘Clinical Neurophysiology, Oulu University Hospital, 90029 OYS, Oulu, Finland’ and Johanna Annunen and Hanna Ansakorpi were incorrectly affiliated with ‘Research Unit of Clinical Neuroscience, Neurology, University of Oulu, 90029 Oulu, Finland.’

The corrected affiliations are as follows:

Ahmed Elabasy^1,3*^, Mia Suhonen^2,3*^, Zalan Rajna^1,3^, Youssef Hosni^1,3^, Janne Kananen^2,3,4^, Johanna Annunen^5,6^, Hanna Ansakorpi^5^, Vesa Korhonen^2,3^, Tapio Seppänen^1^, Vesa Kiviniemi^2,3,7*^

^1^Center for Machine Vision and Signal Analysis, University of Oulu, 90014 Oulu, Finland.

^2^Diagnostics, Medical Research Center, Oulu University Hospital, 90029 Oulu, Finland.

^3^Oulu Functional NeuroImaging, Research Unit of Health Science and Technology, University of Oulu, 90029 Oulu, Finland.

^4^Clinical Neurophysiology, Research Unit of Health Science and Technology, University of Oulu, 90029 Oulu, Finland.

^5^Research Unit of Clinical Neuroscience, Neurology, University of Oulu, 90029 Oulu, Finland.

^6^Neurocenter (Member of ERN EpiCARE), Medical Research Center, Oulu University Hospital, 90029 Oulu, Finland.

^7^Biocenter Oulu, University of Oulu, 90014 Oulu, Finland.

The original Article has been corrected.

