# Supplementary material for: Respiratory brain impulse propagation in focal epilepsy
Source: Sci Rep. 2023 Mar 30;13:5222. doi: 10.1038/s41598-023-32271-7 (PMC10063583; doi:10.1038/s41598-023-32271-7)
Supplement: Supplementary file 1 — Supplementary Information. [file 41598_2023_32271_MOESM1_ESM.docx]

**Supplementary material**

*
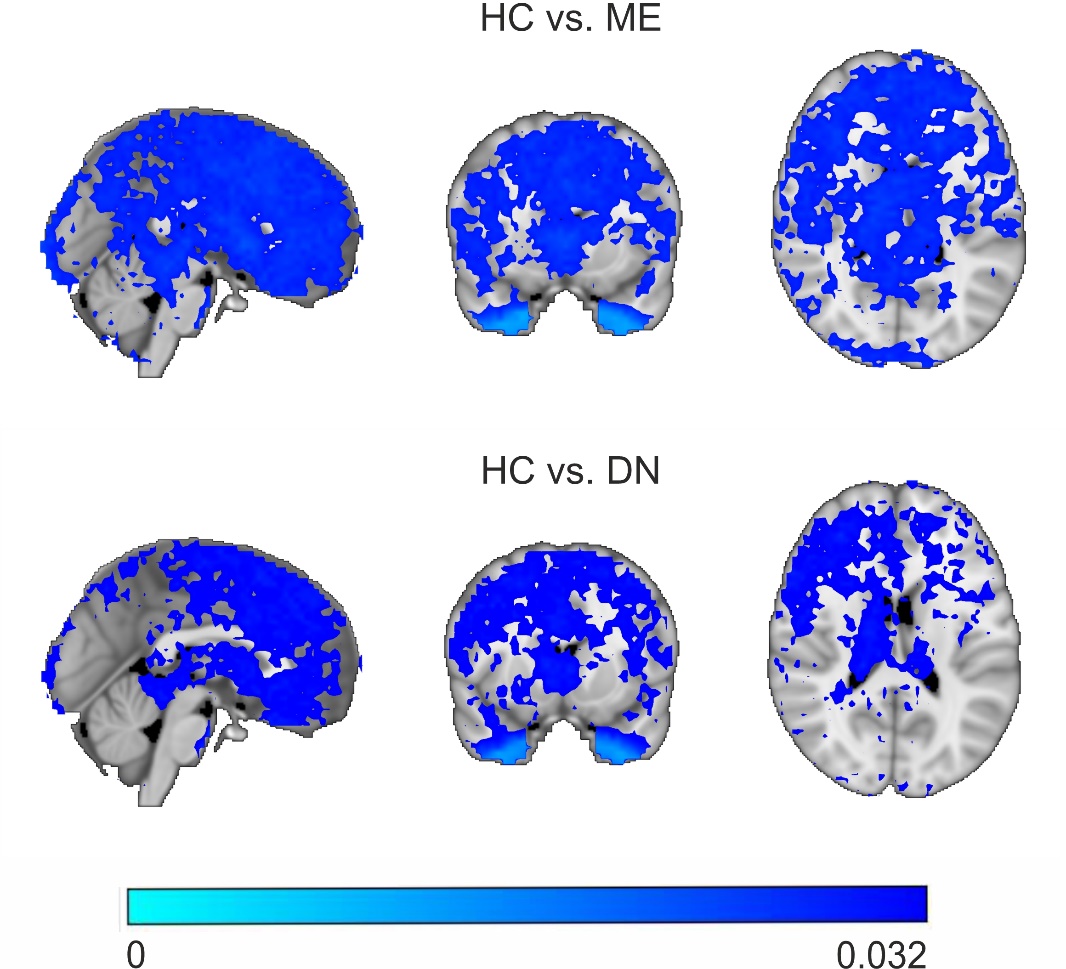
*

**Supplementary Figure 1 Mutual Information of the resampled mean Vresp in comparison between ME (*n*=23) and DN (*n*=19) subjects relative to HC (*n*=75) subjects.** The ME group is more dissimilar/independent compared to HC than are the DN subjects.

**Anatomical details of the V_resp_ in the brain**

The **v_s_** is highest in the brain edges and decreases centripetally because of the opposing motion directions, which are a general property of fluids. We came to understand that the MREG BOLD signal registers the motion of CSF and venous blood flow. The two flows affect each other in the manner described in the methods section. In this study, certain observations confirmed our previous work. In Supplementary Fig. 2A, we show monitoring of the CSF motion direction between the two cerebral hemispheres. In Supplementary Fig. 2B, the **v_s_** of V_resp_ is seen to decrease centripetally in every direction, as if they were two opposing fluid waves. In Supplementary Fig. 2C, we show that the net brain fluid motion is upward, as previously noted^13^. Also, as the respiratory signal propagates through the brain driving the fluid to one side, there is a compensatory motion in the opposite direction, for example as in Supplementary Fig. 2C. This set of observations suggests that the respiratory bandpassed MREG BOLD signal is not noise and is not related directly to neuronal firing.


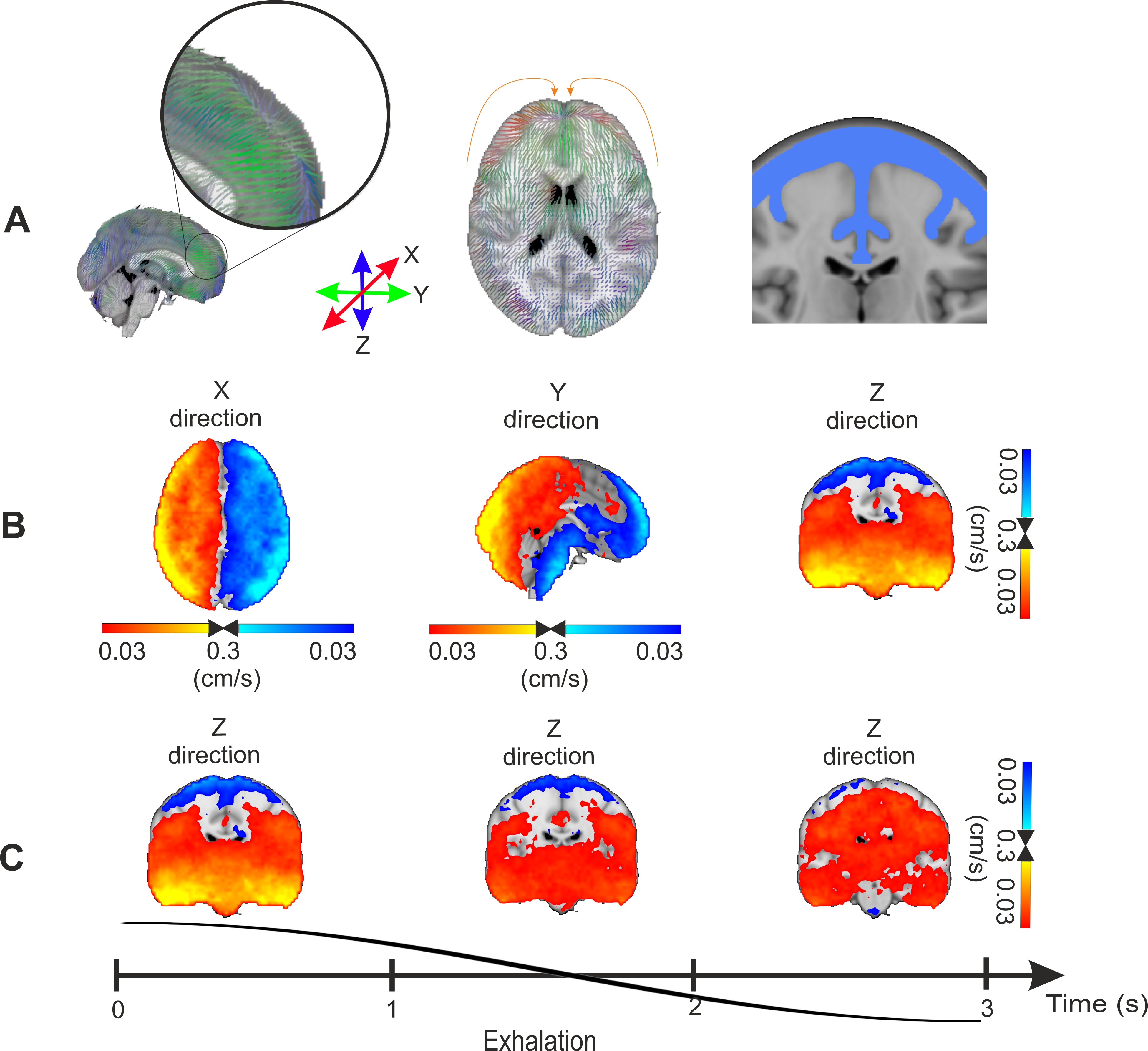


**Supplementary Figure 2 Anatomical details of the mean V_resp_ wave extracted using the optical flow analysis from the HC group (*n*=75).** These details refer to fluid propagating waves affected by respiration. **(A)** the direction of the respiratory wave propagation is in line with the CSF flow in the subarachnoid space between the arachnoid and pia mater, as seen in the coronal anatomical brain view, which exists between the two cerebral hemispheres and around the cerebral cortex. **(B)** the **v_s_** decreases centripetally in every direction, which is a general property of fluids. **(C)** the net **v** (the Z-direction during inhalation and exhalation generally similar, c.f. Fig. 2) is upwards, and the net direction of the CSF flow is upwards (Dreha-Kulaczewski et al., 2017).


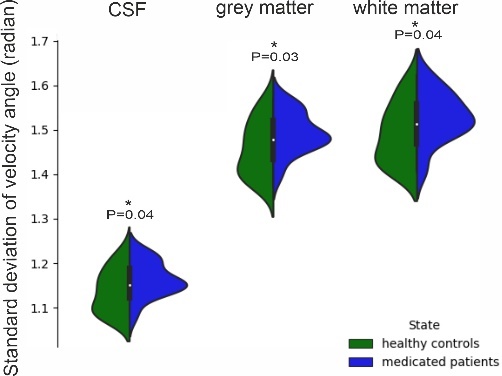


**Supplementary Figure 3 STD in v between ME and HC.** The ME group (*n*=23) have significantly (unpaired two-sample t-test, *P*<0.05) more deviation than the mean flow directionality measured in HC (*n*=75).

**Supplementary Figure 4 Study flow chart.**

**Supplementary Videos 1-6**

[Supplementary video 1 | Optical flow analysis to produce mean V](https://drive.google.com/file/d/1-cC3baGkLr99j_nPlWqYQlATHv1slPPz/view?usp=share_link)_[resp](https://drive.google.com/file/d/1-cC3baGkLr99j_nPlWqYQlATHv1slPPz/view?usp=share_link)_ [maps over an entire respiratory cycle with separated directions.](https://drive.google.com/file/d/1-cC3baGkLr99j_nPlWqYQlATHv1slPPz/view?usp=share_link)

[Supplementary video 2](https://drive.google.com/file/d/1AaRgCk-L64IDafb17CKehgNp8Dz9Jr3K/view?usp=share_link) | Optical flow analysis of HC, ME, and DN.

[Supplementary video 3](https://drive.google.com/file/d/10Cbuwp_IYMruYDDxoUk7cs2UUR-9mzWG/view?usp=share_link) | **v_s_** comparison between ME and HC.

[Supplementary video 4](https://drive.google.com/file/d/1Uxj10aNtpgVlZYoRQG4c0K4C68OoZo_O/view?usp=share_link) | **v** comparison between ME and HC.

[Supplementary video 5](https://drive.google.com/file/d/1F9E-gy31E-siSDgKciiS7rIHZVuX2DoI/view?usp=share_link) | **v_s_** comparison between DN and HC.

[Supplementary video 6](https://drive.google.com/file/d/1jQ5dI2K1pWZCe6KCXIBS9GRyyNtx0PmK/view?usp=share_link) | **v** comparison between DN and HC.

[Supplementary video 7](https://drive.google.com/file/d/1D7JrZ6yp2c7xqF6SOaZ_tfSRY2g_U3Or/view?usp=share_link) | Optical flow analysis of 23 HC and the 23 ME.
